# Glymphatic dysfunction as a predictor of response to lacosamide add-on therapy in drug-resistant focal epilepsy

**DOI:** 10.3389/fneur.2026.1749772

**Published:** 2026-03-06

**Authors:** Dong Ah Lee, Jin-Hong Wi, Ho-Joon Lee, Kang Min Park

**Affiliations:** 1 Department of Neurology, Haeundae Paik Hospital, Inje University College of Medicine, Busan, Republic of Korea; 2 Department of Thoracic and Cardiovascular Surgery, Busan Paik Hospital, Inje University College of Medicine, Busan, Republic of Korea; 3 Department of Radiology, Haeundae Paik Hospital, Inje University College of Medicine, Busan, Republic of Korea

**Keywords:** anticonvulsants, diffusion tensor imaging, epilepsy, glymphatic system, lacosamide

## Abstract

**Background:**

Drug-resistant epilepsy continues to be a major challenge, as treatment response to additional antiseizure medications is often limited, and early prediction remains crucial. The diffusion tensor image analysis along the perivascular space (DTI-ALPS) index has emerged as a noninvasive imaging method to assess glymphatic function, which plays a key role in brain waste clearance. We aimed to evaluate whether the DTI-ALPS index could serve as a valuable prognostic biomarker of treatment response in patients with drug-resistant focal epilepsy receiving lacosamide (LCM) add-on therapy.

**Methods:**

We retrospectively enrolled 155 patients with drug-resistant focal epilepsy who underwent diffusion tensor imaging (DTI) prior to initiating LCM add-on therapy and had at least 12 months of clinical follow-up. Patients were classified into LCM responders and non-responders based on seizure reduction. The DTI-ALPS index was calculated from preprocessed DTI data acquired on a 3 T magnetic resonance imaging scanner and compared between the groups.

**Results:**

Among 155 patients with drug-resistant focal epilepsy, 33 were classified into LCM responders and 122 into LCM non-responders. Non-responders had a higher number of prior antiseizure medication (ASM) burden (3 vs. 2, *p* < 0.001) and more frequent epileptiform discharges on electroencephalography (78.5% vs. 57.6%, *p* = 0.015). Additionally, the DTI-ALPS index was significantly greater in responders (1.4022 vs. 1.1936, *p* = 0.024) than in non-responders, and receiver operating characteristic curve analysis showed its predictive value for LCM response (area under the curve = 0.620, *p* = 0.015).

**Conclusion:**

DTI-ALPS index was significantly lower in non-responders to LCM add-on therapy among patients with drug-resistant focal epilepsy, suggesting that glymphatic dysfunction may contribute to reduced ASM responsiveness and serve as a potential noninvasive biomarker to aid in treatment prediction.

## 1 Introduction

Among the approximately 65 million people with epilepsy worldwide (1), about 60% experience focal seizures (2), and predicting treatment response and drug resistance to antiseizure medication (ASM) remains a major challenge. Drug-resistant epilepsy (DRE)—also referred to as refractory, intractable, or pharmacoresistant epilepsy—accounts for a substantial proportion of this burden and continues to be one of the most pressing issues in epilepsy care. The International League Against Epilepsy Commission on Therapeutic Strategies proposed a consensus operational definition of DRE in 2009, as “failure of adequate trials of two tolerated, appropriately chosen, and adequately used antiepileptic drug schedules (whether as monotherapies or in combination) to achieve sustained seizure freedom.” (3) If seizures are not controlled with the first administered ASM, the probability of achieving seizure freedom with subsequent pharmacological interventions is substantially diminished. Accordingly, the initial therapeutic response has been recognized as a critical prognostic determinant. Moreover, patients with symptomatic or cryptogenic etiologies exhibit a higher risk of developing DRE, and unfavorable outcomes are well-documented in those with structural abnormalities such as mesial temporal sclerosis or cortical dysplasia, as well as in patients presenting with a high seizure frequency prior to the initiation of treatment (3, 4).

Recent studies have shown that in individuals with newly diagnosed focal epilepsy who initiated ASM therapy within 4 months of diagnosis, 54.7% were treatment sensitive, most of whom achieved seizure control with monotherapy. However, more than half of these patients required over 1 year and more than two ASM trials to achieve seizure freedom. In newly diagnosed focal epilepsy, DRE is often identified within the first 6–8 months of treatment. The response to ASM is strongly influenced by baseline clinical characteristics, as patients with psychiatric comorbidities or higher pretreatment seizure frequencies tend to show lower treatment sensitivity and higher resistance. These factors are also associated with reduced treatment retention and may necessitate careful consideration of potential drug–drug interactions (5).

Early prediction of DRE and timely identification of patients who may require treatment beyond medication are important clinical decisions. Many studies have attempted to develop predictive models using brain magnetic resonance imaging (MRI) and electroencephalography (EEG) data (6–9), and more recently, big data-driven machine learning approaches incorporating genomic information, as well as advanced neuroimaging data, have been increasingly investigated (10–12). Among these, brain MRI data have accumulated the most substantial evidence for predicting DRE, with various methods proposed, particularly those using diffusion tensor imaging (DTI) (9, 13, 14).

Taoka et al. introduced the diffusion tensor image analysis along the perivascular space (DTI-ALPS) method, which enables the evaluation of glymphatic function with a single MRI acquisition (15). The glymphatic system is a concept of central nervous system (CNS) interstitial fluidopathy, representing the flow of cerebrospinal fluid (CSF) within the brain and functioning as the waste clearance system of the CNS (16–18). To calculate the DTI-ALPS index, regions of interest (ROIs) are placed near the body of the lateral ventricle, where projection fibers, association fibers, and medullary veins run orthogonally. This anatomical configuration minimizes the influence of water diffusivity along major white matter tracts, thereby allowing a more specific assessment of diffusivity along the perivascular space. Diffusivities in each corresponding direction are obtained from DTI (15). A recent study validated the DTI-ALPS index against glymphatic MRI with intrathecal gadolinium administration, demonstrating that it is a reliable non-invasive marker of glymphatic clearance function (19). Recent studies have highlighted that dysfunction of the glymphatic system is associated with various neurological disorders, raising the possibility of its use as a biomarker for early diagnosis, disease progression assessment, and monitoring of therapeutic efficacy (20).

This study aimed to evaluate whether alterations in the glymphatic system of patients with DRE could serve as a predictor of response to subsequent add-on therapy using the DTI-ALPS index.

## 2 Methods

### 2.1 Participants

The protocol for this retrospective study, conducted at a single university hospital, was approved by the institutional review board. Participants were identified from the epilepsy database of our institution based on the following inclusion criteria: (1) patients with focal epilepsy diagnosed on the basis of seizure semiology and EEG findings (21), (2) patients whose symptoms fulfill the definition of DRE (failure of adequate trials of two tolerated, appropriately chosen, and adequately used ASM regimens to achieve sustained seizure freedom) (3), (3) patients who underwent DTI MRI as part of the routine epilepsy MRI protocol prior to the initiation of lacosamide (LCM) add-on therapy, and (4) patients with a minimum of 12 months of regular follow-up after the initiation of LCM add-on therapy.

We collected data on demographic and clinical characteristics, including age, sex, family history of epilepsy, age at seizure onset, prior ASM burden, duration of follow-up period, and the presence of abnormalities on brain MRI and EEG. Additionally, we classified patients with focal epilepsy and DRE who received LCM add-on therapy into responder and non-responder groups according to their treatment response. LCM responders were defined as patients who maintained seizure freedom throughout the follow-up period after the initiation of LCM add-on therapy (22–24).

### 2.2 Acquisition of DTI

All MRI examinations were performed on the same 3.0 T scanner (AchievaTx, Philips Healthcare, Best, The Netherlands) equipped with a 32-channel head coil. All patients with drug-resistant focal epilepsy underwent the same brain MRI protocol before add-on treatment with LCM. The protocol included 3-dimensional (3D) fluid-attenuated inversion recovery imaging, coronal T2-weighted imaging, 3D T1-weighted imaging, and DTI. DTI was acquired using a spin-echo, single-shot echo-planar imaging sequence with 32 diffusion-encoding directions (repetition time/echo time = 8620/85 ms, flip angle = 90°, slice thickness = 2.25 mm, acquisition matrix = 120 × 120, field of view = 240 × 240 mm^2^, and b-value = 1,000 s/mm^2^).

### 2.3 Calculation of the DTI-ALPS index

DTI preprocessing was performed using DSI Studio.^1^ The preprocessing steps included acquisition of open-source images, correction for eddy current and phase-distortion artifacts, generation of a brain mask (thresholding, smoothing, and defragmentation), and reconstruction with the DTI method (25, 26).

A rectangular ROI was placed at the level of the lateral ventricle body. Within this ROI, fiber orientation and diffusivities along the x-, y-, and z-axes were obtained at the voxel level. For each fiber type (projection, association, and subcortical fibers along the x-axis), the voxel showing the maximum orientation was selected. The DTI-ALPS index was then calculated using the following formula (15, 27, 28):


$$\mathrm{DTI}-\text{ALPS index}=\frac{\text{mean}\;(\text{Dxxproj},\text{Dxxassoci})}{\text{mean}\;(\text{Dyyproj},\text{Dzzassoci})}$$

**Figure 1:**
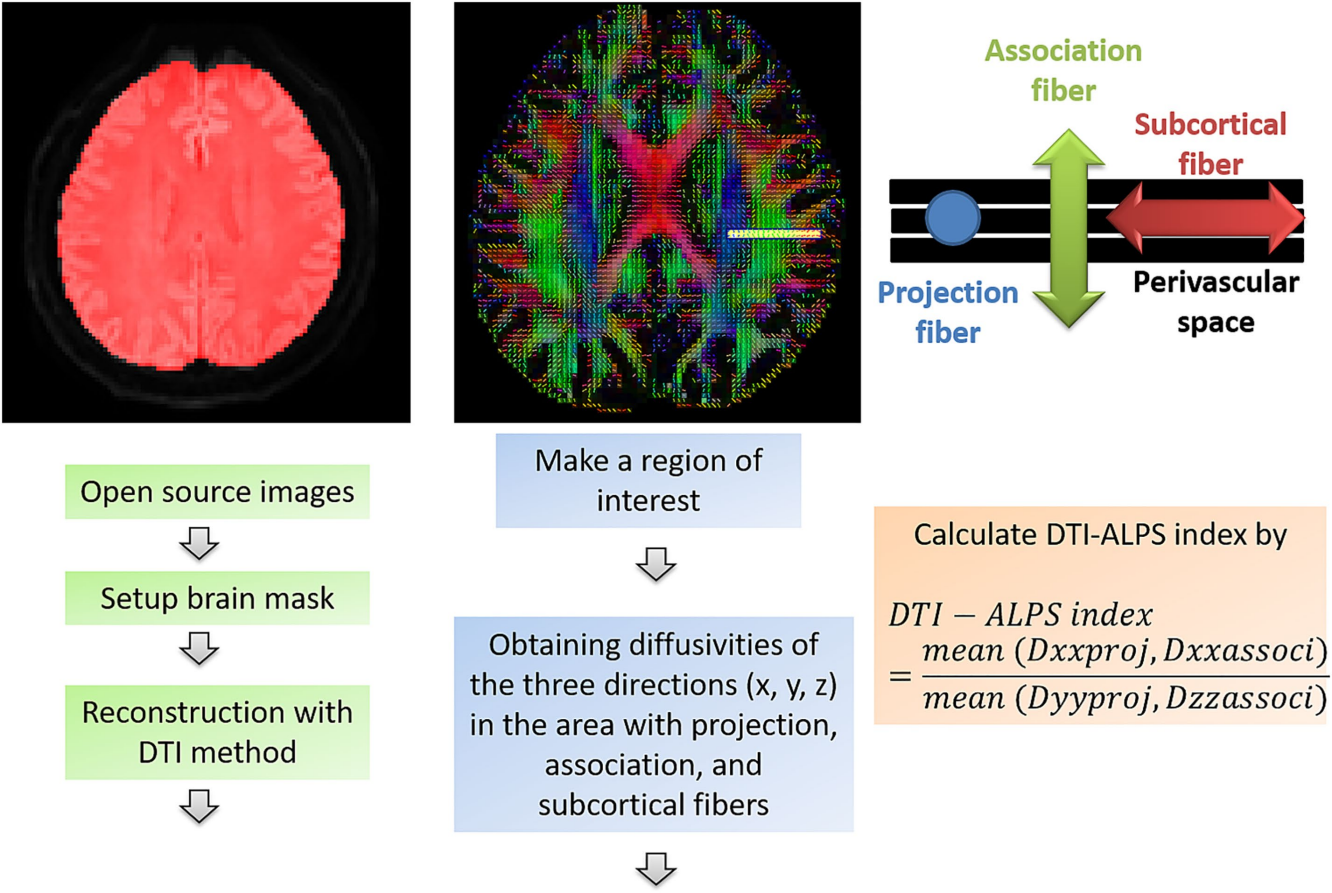
Workflow for calculating the DTI-ALPS index in this study. DTI-ALPS index: diffusion tensor image analysis along the perivascular space index, D_xx_proj: diffusivity along the *x*-axis in the projection fiber, D_xx_associ: diffusivity along the *x*-axis in the association fiber, D_yy_proj: diffusivity along the *y*-axis in the projection fiber, D_zz_associ: diffusivity along the *z*-axis in the association fiber.

Dxxproj: diffusivity along the x-axis in the projection fiber, Dxxassoci: diffusivity along the x-axis in the association fiber, Dyyproj: diffusivity along the y-axis in the projection fiber, Dzzassoci: diffusivity along the z-axis in the association fiber. A schematic overview of the calculation process for the DTI-ALPS index is shown in Figure 1.

### 2.4 Statistical analysis

The primary analyses were based on *a priori*-specified hypotheses. No formal statistical power calculation was performed in advance, as this was a retrospective study, and the sample size was determined by the available data. A post-hoc power analysis was conducted to assess the statistical power of the primary outcome given the final sample size and observed effect size. Differences in demographic and clinical characteristics between groups were assessed using an independent-samples t-test or Mann–Whitney U test, depending on the distribution of continuous variables, and the Chi-square test for categorical variables. Intergroup comparisons of diffusivity measures and the DTI-ALPS index were performed using independent-samples t-tests. Receiver operating characteristic (ROC) curve analysis was performed to evaluate the predictive power of the DTI-ALPS index for LCM response and to determine the optimal cutoff. Categorical variables were summarized as frequencies and percentages, and continuous variables as mean ± standard deviation or median with interquartile range. Statistical significance was set at a two-tailed *p*-value <0.05. To identify independent predictors of LCM response, a multivariable logistic regression analysis was performed using the backward elimination method. Variables with a *p*-value < 0.05 in the univariate analysis were included in the model. All analyses were conducted using MedCalc® Statistical Software, version 20.014.^2^

## 3 Results

### 3.1 Demographic and clinical characteristics of patients with drug-resistant focal epilepsy

We enrolled 155 patients with drug-resistant focal epilepsy. Table 1 shows the demographic and clinical characteristics of patients. Of the 155 patients with drug-resistant focal epilepsy, 33 patients showed good responses and 122 patients showed poor responses to add-on LCM treatment. Age, sex, family history, duration of follow-up period, and presence of MRI abnormalities did not differ between LCM responders and non-responders. However, non-responders had an earlier age of seizure onset (18 vs. 24 years, *p* = 0.039), a higher number of prior ASM burden (3 vs. 2, *p* < 0.001) and more frequent epileptiform discharges on EEG than LCM responders (78.5% vs. 57.6%, *p* = 0.015). No significant difference was identified in the prior use of sodium channel-blocking agents (69.7% vs. 84.4%, *p* = 0.055).

**Table 1:** Comparison of demographic and clinical characteristics between LCM responders and non-responders among patients with drug-resistant focal epilepsy.

	LCM responders (*n* = 33)	LCM non-responders (*n* = 122)	*p*-value
Age, years (±SD)	41.4 (±15.7)	36.8 (±13.1)	0.095
Male, *n* (%)	13 (39.4)	56 (45.9)	0.505
Family history, *n* (%)	0 (0)	9 (7.4)	0.450
Age of seizure onset, years (interquartile range)	24 (14–42)	18 (10–25)	0.039
Duration of follow-up after LCM added, months (interquartile range)	64 (30–76)	65 (33–79)	0.638
Prior ASM burden (interquartile range)	2 (2–3)	3 (2–4)	<0.001
Prior use of blocking Na channel, *n* (%)	23 (69.7%)	103 (84.4)	0.055
MRI abnormality, *n* (%)	21 (63.6)	82 (67.8)	0.656
Epileptiform discharges on EEG, *n* (%)	19 (57.6)	95 (78.5)	0.015

n: number, LCM: lacosamide, SD: standard deviation, ASM: anti-seizure medication, Na: sodium, EEG: electroencephalography, MRI: magnetic resonance imaging.

### 3.2 Differences in the DTI-ALPS index between LCM responders and non-responders among patients with drug-resistant focal epilepsy

**Figure 2:**
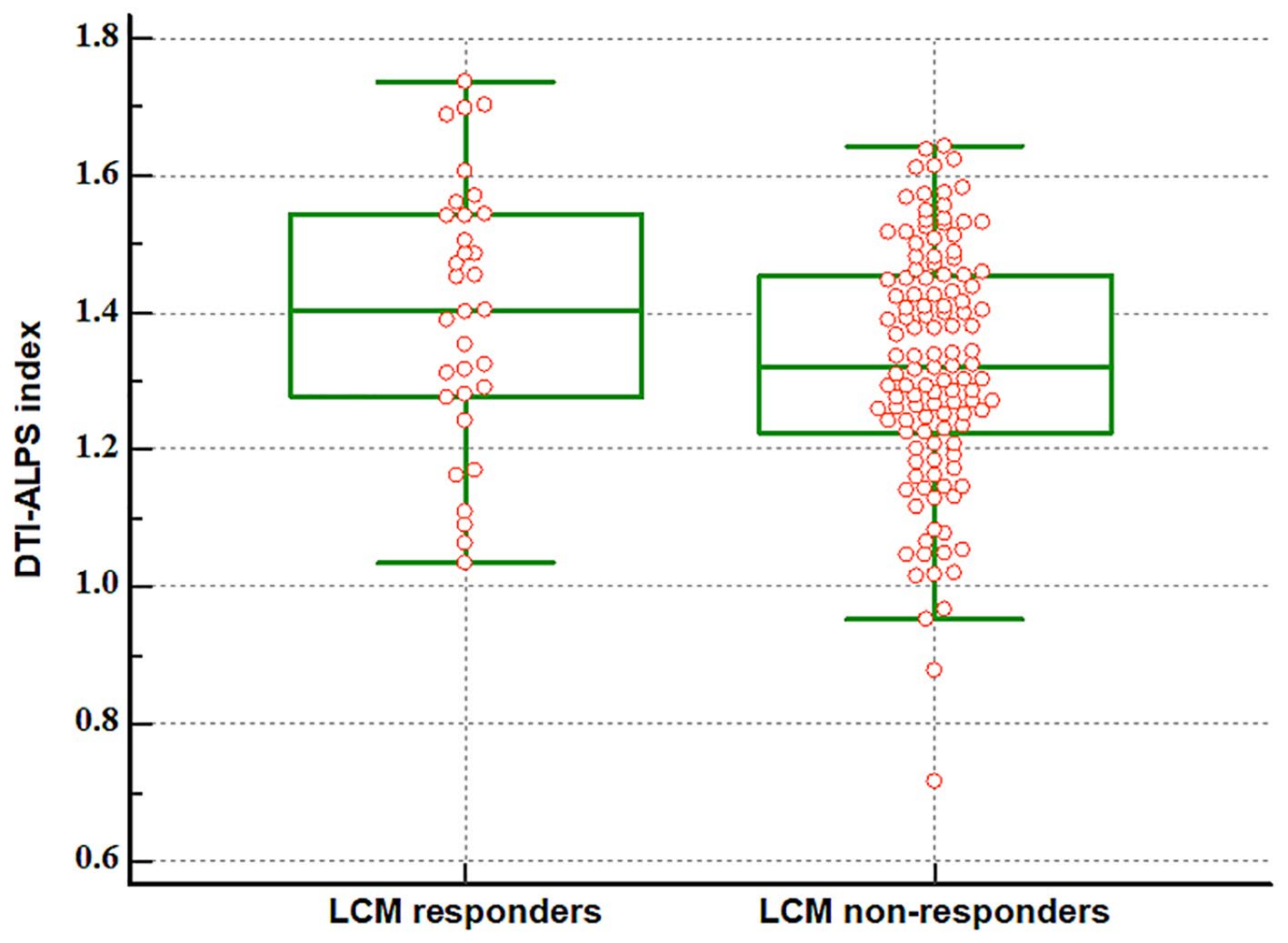
Difference in DTI-ALPS index between LCM responders and non-responders among patients with drug-resistant focal epilepsy. The DTI-ALPS index was significantly lower in non-responders compared to responders among patients with drug-resistant focal epilepsy (1.1936 vs. 1.4022, *p* = 0.024). DTI-ALPS index: diffusion tensor image analysis along the perivascular space index, LCM: lacosamide.

The DTI-ALPS index differed significantly between LCM responders and non-responders among patients with DRE (Table 2, Figure 2) (1.4022 vs. 1.3218, *p* = 0.024). Furthermore, diffusivity along the y-axis in the projection fibers showed a significant difference between LCM responders and non-responders among patients with drug-resistant focal epilepsy (0.00049 vs. 0.00053, *p* = 0.040).

**Table 2:** The differences in diffusivities and DTI-ALPS indices between LCM responders and non-responders among patients with drug-resistant focal epilepsy.

	LCM responders (*n* = 33)		LCM non-responders (*n* = 122)		*p*-value
	Mean	SD	Mean	SD	
Projection fiber					
Dxx	0.00058	0.00001	0.00058	0.00000	0.669
Dyy	0.00049	0.00001	0.00053	0.00011	0.040
Dzz	0.00102	0.00001	0.00103	0.0000	0.641
Association fiber					
Dxx	0.00064	0.00000	0.00066	0.00001	0.375
Dyy	0.00101	0.00001	0.00108	0.00011	0.474
Dzz	0.00039	0.00009	0.00042	0.00008	0.093
Subcortical fiber					
Dxx	0.00108	0.00013	0.00108	0.00012	0.635
Dyy	0.00068	0.00011	0.00070	0.00015	0.450
Dzz	0.00060	0.00015	0.00060	0.00014	0.972
DTI-ALPS index	1.4022	0.1936	1.3218	0.1761	0.024

n: number, DTI-ALPS index: diffusion tensor image analysis along the perivascular space index, LCM: lacosamide, SD: standard deviation, Dxx: diffusivity along the x-axis, Dyy: diffusivity along the y-axis, Dzz: diffusivity along the z-axis.

### 3.3 DTI-ALPS index in predicting LCM response

**Figure 3:**
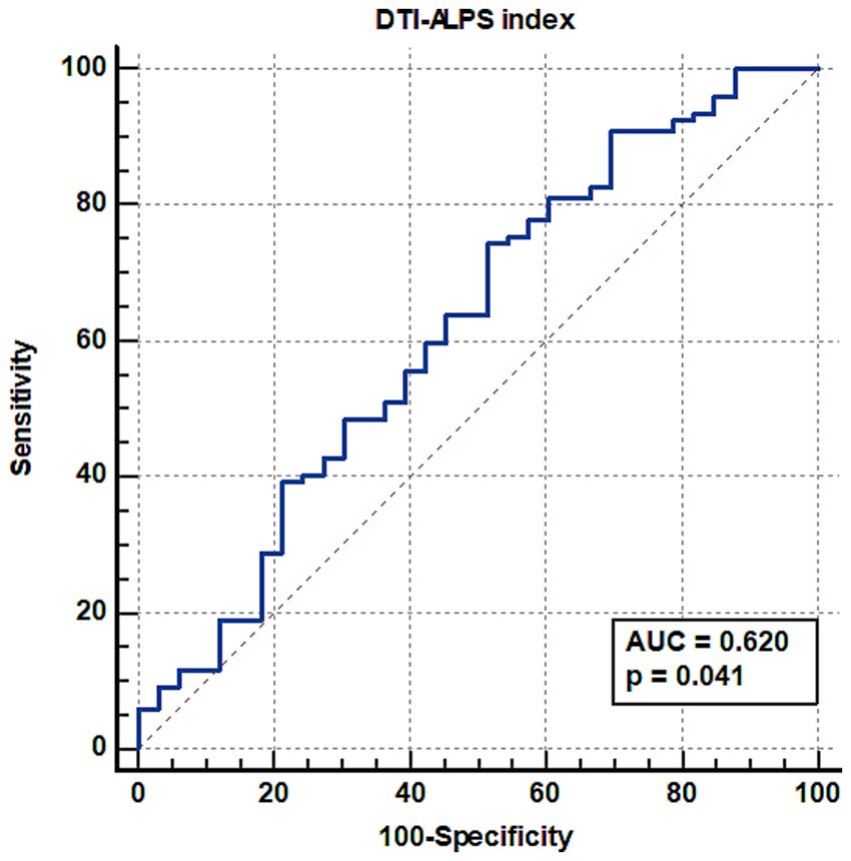
Receiver operating characteristic curve analysis of the DTI-ALPS index for predicting LCM response in patients with drug-resistant focal epilepsy. The DTI-ALPS index predicted response to add-on LCM treatment, with an area under the curve (AUC) of 0.620. DTI-ALPS index: diffusion tensor image analysis along the perivascular space index, LCM: lacosamide.

Figure 3 shows the ROC curve analysis of the DTI-ALPS index for predicting LCM response. In patients with DRE, the DTI-ALPS index predicted the drug response to add-on LCM (area under the curve = 0.620, *p* = 0.015).

### 3.4 Multivariable analysis for predictors of LCM response

We performed a multivariable logistic regression analysis including age of onset, prior ASM burden, epileptiform discharges on EEG, and the DTI-ALPS index (Table 3). The analysis identified prior ASM burden (*p* = 0.003) and epileptiform discharges on EEG (*p* = 0.027) as independent predictors of poor treatment response. Age of seizure onset was not statistically significant in the multivariable model (*p* = 0.168) The DTI-ALPS index showed a trend toward significance (*p* = 0.096) but did not remain statistically significant after adjusting for clinical covariates.

**Table 3:** Multivariable logistic regression analysis of factors associated with LCM non-responder.

	Coefficient	Odds ratio	95% CI	*p*-value
Age of seizure onset	−0.021	0.980	0.951–1.009	0.168
Prior ASM burden	0.892	2.440	1.366–4.359	0.003
Epileptiform discharges on EEG	−1.055	0.348	0.137–0.888	0.027
DTI-ALPS index	−2.207	0.110	0.008–1.479	0.096

## 4 Discussion

The main finding of this study is that LCM responders exhibited significantly higher DTI-ALPS index compared with non-responders. These results suggest that impaired glymphatic function, as reflected by the lower DTI-ALPS index, may be associated with poor therapeutic response to LCM in patients with DRE, highlighting the potential utility of the DTI-ALPS index as a surrogate marker for guiding individualized treatment strategies.

LCM exhibited a unique antiseizure mechanism by selectively enhancing the slow inactivation of voltage-gated sodium channels, thereby stabilizing neuronal membrane excitability and reducing pathologic hyperexcitability underlying seizure generation (22–24). In three pivotal randomized controlled trials, adjunctive LCM at 400 mg/day achieved ≥50% responder rates of 38, 40.5, and 41% across the studies, with seizure freedom achieved in only a small proportion of patients. These modest outcomes reflected the highly refractory nature of the trial populations, who had long disease duration and multiple prior treatment failures (22–24). Real-world studies have reported substantially higher efficacy of adjunctive LCM when introduced earlier in the treatment course. In patients who were generally less treatment-refractory and received LCM as an add-on to a single baseline ASM, 72.5% achieved ≥50% seizure reduction and 45.5% attained seizure freedom after 6 months. These findings suggest that the clinical benefit of LCM may be more pronounced in routine practice, particularly when used early (29). The results of this study demonstrated lower efficacy of adjunctive LCM compared with previous real-world reports (29). In our cohort, only 21% of patients achieved a ≥ 50% reduction in seizure frequency. The cohort had greater prior ASM exposure, typically 2–4 medications, with structural abnormalities on MRI in 66% of patients and epileptiform discharges on EEG in 74%, indicating a more refractory clinical profile. Moreover, the present analysis had a long-term follow-up, with a median LCM retention of 64 months, whereas earlier studies generally reported short-term outcomes of 6–12 months, where efficacy rates were typically higher.

The mechanisms of glymphatic dysfunction in epilepsy have been described in previous studies, and at the molecular and cellular level, seizure activity induces redistribution and functional impairment of astrocytic aquaporin-4, thereby disrupting the exchange between CSF and interstitial fluid and reducing the clearance of metabolic waste products (30). Additionally, seizure-related disruption of the blood–brain barrier allows serum proteins, inflammatory cytokines, and immune cells to infiltrate the parenchyma, occluding the perivascular spaces and hindering glymphatic flow. Activated microglia further exacerbate this pathological state by releasing proinflammatory cytokines, including interleukin-1β and tumor necrosis factor-*α*, which enhance neuronal excitability and diminish clearance efficiency (30). Specifically, in patients with temporal lobe epilepsy with hippocampal sclerosis, gliosis leads to a localized collapse and reduction of perivascular spaces, whereas in the acute postictal phase, phosphorylated tau can accumulate within the perivenous space, resulting in a transient expansion of these channels (31). Furthermore, epilepsy frequently disrupts sleep architecture, particularly slow-wave sleep, which is essential for efficient glymphatic clearance. Seizure activity and interictal discharges fragment sleep and reduce time spent in restorative stages, thereby impairing the nocturnal removal of neurotoxic substances (30). Vascular comorbidities such as cerebral small vessel disease may further exacerbate these disturbances by reducing the arterial pulsatility required to drive cerebrospinal fluid into the perivascular pathways (30). Glymphatic dysfunction was found to be more pronounced with longer seizure duration. The number of perivascular spaces may decrease over time following seizure onset. This phenomenon could reflect the reversibility of blood–brain barrier disruption and a compensatory stage of the glymphatic system, suggesting a partial restoration of its function after the acute phase (32, 33). MRI-based studies have demonstrated that the number and size of Virchow–Robin spaces are increased in patients with epilepsy (33). Previous investigations using the DTI-ALPS method have also reported a reduction of the DTI-ALPS index across different epilepsy subtypes, thereby providing imaging evidence of glymphatic dysfunction in epilepsy (34–37).

Early identification of DRE represents a pivotal element in optimizing treatment strategies for patients with epilepsy. A range of advanced neuroimaging methodologies, including analyses of structural connectivity, functional connectivity, and structural covariance derived from various MRI sequences, have been proposed to elucidate factors associated with ASM response (14, 38, 39). In line with these approaches, our previous investigations demonstrated that patterns of functional covariance networks differed according to ASM response in patients with newly diagnosed epilepsy (8). Furthermore, a study focusing on patients receiving levetiracetam as first-line monotherapy highlighted structural connectivity as a potential predictive factor for treatment responsiveness (40). Likewise, an add-on study with perampanel (PER) further demonstrated that patients with fewer prior ASM exposures were more likely to respond favorably, with structural connectivity differing according to responsiveness to adjunctive PER treatment (41). Taken together, these findings reinforce the role of network-based imaging markers in predicting ASM response and guiding early therapeutic decisions.

In our previous study analyzing patients with focal epilepsy who exhibited no visible lesions on brain MRI, the DTI-ALPS index was significantly lower in the group showing poor response to ASM and demonstrated a negative correlation with both age and duration of epilepsy (28). Given that the delivery of ASM to neurons is closely dependent on glymphatic system flow, dysfunction of this system may be directly associated with diminished responsiveness to pharmacological treatment (42). The present findings are consistent with this concept, suggesting that patients with impaired glymphatic clearance may have reduced drug bioavailability within the brain parenchyma, leading to insufficient pharmacological efficacy even when appropriate ASMs are prescribed. Additional mechanisms may explain why our study demonstrated lower DTI-ALPS indices in LCM non-responders. First, chronic seizure activity can exacerbate glymphatic dysfunction through repeated astrocytic and vascular changes, thereby compounding resistance to treatment. Second, patients with longer epilepsy duration and greater prior ASM burden—both of which were more prevalent in our non-responder group—are more likely to exhibit progressive impairment of glymphatic transport, as suggested by experimental and imaging studies. Third, LCM’s mechanism of action, which primarily involves modulation of sodium channel slow inactivation, may be particularly sensitive to efficient drug delivery and homeostatic clearance processes; disruption of perivascular transport could therefore blunt its therapeutic impact. Consistently, in our multivariable model, the DTI-ALPS index did not maintain independent statistical significance (*p* = 0.096) when adjusting for potent clinical predictors such as prior ASM burden and EEG findings. This finding aligns with the modest discriminative performance observed in our ROC analysis (AUC = 0.620), suggesting that the DTI-ALPS index alone is insufficient to serve as a definitive standalone clinical predictor. Instead, these results support the notion that glymphatic dysfunction is closely intertwined with disease severity. Therefore, the DTI-ALPS index functions as a valuable biological surrogate that reflects the cumulative burden of epilepsy, acting as a complementary biomarker to established clinical parameters rather than replacing them.

Recently, machine learning models have been applied to predict ASM responsiveness, showing encouraging accuracy (11). Nonetheless, their clinical translation remains limited due to the restricted availability of variables such as genetic or multimodal datasets. Contrastingly, glymphatic system assessment may provide a more intuitive and clinically accessible tool for predicting treatment response, underscoring its potential relevance as a biomarker in therapeutic decision-making.

This study has some limitations. First, it was a retrospective, single-center study with a relatively small responder group (*n* = 33), which may limit statistical power and precision. Second, we adopted a stringent definition of “LCM responder,” requiring complete seizure freedom. This decision was made to ensure the accuracy of the clinical outcome, as retrospective quantification of seizure reduction (e.g., ≥50%) is often limited by recall bias and incomplete documentation. While this rigorous approach ensures a reliable classification of responders, it may have underestimated the broader therapeutic benefits of LCM, as patients achieving meaningful seizure reduction without full remission were classified as non-responders. Third, given that ASM responsiveness can evolve dynamically over time, we could not adequately capture delayed or fluctuating treatment responses. Additionally, sleep architecture represents an unmeasured confounder, as glymphatic activity is maximized during slow-wave sleep and may be functionally suppressed in patients with drug-resistant epilepsy who experience significant sleep fragmentation. Although prior studies suggest that the DTI-ALPS index remains stable during the awake state and all MRI scans were performed during routine daytime hours (43, 44), the absence of objective sleep quality measures remains a limitation (45). Collectively, these limitations warrant caution in interpreting our results and highlight the necessity of future prospective, multicenter investigations with larger cohorts, longitudinal assessments, and integration of sleep-related measures to validate and extend these observations.

## 5 Conclusion

The DTI-ALPS index was significantly lower in non-responders to LCM add-on therapy among patients with drug-resistant focal epilepsy, suggesting that glymphatic dysfunction is associated with reduced ASM responsiveness and may help identify patients who are unlikely to benefit from additional antiseizure medications.

## Data Availability

The original contributions presented in the study are included in the article/supplementary material, further inquiries can be directed to the corresponding author.

## Footnotes

1 version 2021 May; http://dsi-studio.labsolver.org.

2 MedCalc Software Ltd., Ostend, Belgium, 2021; https://www.medcalc.org.
